# ‘Everyone in my family has C-sections’: increased likelihood of caesarean birth in family lineages in the United States

**DOI:** 10.1093/emph/eoaf018

**Published:** 2025-07-15

**Authors:** Kathleen M Hanlon-Lundberg

**Affiliations:** Department of Anthropology, Department of Public Health, Wayne State University, Detroit, MI, USA

**Keywords:** caesarean, birth, transgenerational, maternal lineages, human reproduction, parity

## Abstract

Caesarean birth has multiple, interrelated, and often mutually reinforcing bio-social etiologies. Evolutionary consequences of caesarean are uncertain. The goal of this study is to determine if caesarean births are more likely within family lineages by connecting and comparing lifetime birth experience (caesarean, vaginal) of individual women and generations of their families. A secondary goal is to identify potential parity differences between birth modes. Qualitative and quantitative methods were used to evaluate women’s birth narratives of their own births and births to their maternal relatives: grandmothers, mothers, sisters, daughters. Participant data was analysed by birth year cohort and by familial generation (Grandmother, Mother, Index, and Daughter). 107 women participated in the study. Parous daughters of women who experienced any caesarean were more likely to experience caesarean than were daughters of women experiencing all vaginal birth (*P* = .0002; relative risk 2.1 [1.53–2.88]). Prevalence of any caesarean per mother is higher than the per-birth caesarean rate (44/107, 41.12% versus 71/229, 31.00%; *P* = .03). Parity was higher for women experiencing all vaginal births than all caesarean (2.14 versus 1.79; *P* = .03), yet highest for those experiencing any caesarean (2.75; *P* = .01). Caesarean for any indication is more common among daughters of mothers who themselves experienced any caesarean than those who experienced all vaginal births. Individual lifetime caesarean experience is more prevalent than commonly construed based on caesarean per live birth rate. Clustering of cesarean within families suggests that operative birth is altering our bio-social selves in potentially heritable ways.

## INTRODUCTION

Surgical birth (caesarean birth, CB) has dramatically increased over the past two to three human generations. Currently, more than one of five births worldwide is via CB; the United States’ CB rate is nearly one in three births [1, 2]. Birth through abdominal incision with or without labor is now the most common surgery in the world [3]. Altering processes that directly influence a population’s reproductive success is likely to influence the evolutionary trajectory of that species. Is caesarean birth producing human lineages which are more likely to utilize or to require surgical birth? Prevalence of heritable characteristics relies on the proportion of surviving progeny who themselves go on to successful reproduction: what is the relationship between CB and parity?

Increasing CB prevalence has multiple, interrelated, and often mutually reinforcing etiologies, including biological (physiologic, anatomic) and sociological (institutional and agentive). Biological factors related to CB include proportionately large fetal heads relative to maternal pelves. The ‘obstetric dilemma’ posits that increasing encephalization during human evolution driving larger fetal head size is limited by maternal pelvic dimensions necessary to support bipedal locomotion [4]. Emergent agricultural practices may have contributed to the challenges of human birth as increasingly adipose babies were born to mothers of relatively smaller stature [5]. Through natural selection, ‘any variation in the least injurious <is> rigidly destroyed’ [6]. Mitteroecker describes a ‘cliff-edge model’ of obstetric selection for fetopelvic disproportion (FPD), including cephalopelvic disproportion and shoulder dystocia [7, 8]. Mathematical modeling indicates that removing pelvic restriction on the upward selective force of larger babies through CB predicts an increase in FPD rates by 10%–20%.

Medical and obstetric indications for CB may be overtly or implicitly conflated with personal, social, institutional, and fiscal considerations which generally favor operative delivery. For example, the diagnosis of FPD may seem straightforward: fetal head dimensions exceed those of the maternal pelvis. FPD may result in non-progressive labor. However, ultrasonically measured fetal dimensions to estimate FPD risk are not accurate predictors of successful vaginal birth (VB) [9]. Unfused fetal cranial plates shift and mold to the maternal pelvis. Physiological changes during pregnancy render maternal pelvis somewhat flexible [10] and functional pelvic dimensions are influenced by position [11]. Uterine contractions effect descent and rotation of the fetus through the maternal pelvis. Difficulty with any of these factors, or combinations thereof, can result in non-progressive labor. How much change over what time period constitutes labor progress? Efforts have been made to assess labor progress in connection with maternal–child outcomes; debate as to guideline validity persists [12, 13].

A multitude of indications for CB expand its use beyond FPD and non-progressive labor. Fetal heart rate monitoring was rapidly implemented in the 1970’s, leading to a rise in diagnoses of fetal distress or labor intolerance, which is now the second most common indication for CB after nonprogressive labor [14, 15]. Approximate 2.5% of term babies are breech; the proportion increases with prematurity [16]. Perinatal morbidity and mortality for breech-presenting fetuses has been demonstrated to be slightly lower for planned CB versus planned VB [17]. Likewise, twin gestation accounts for ~3.4% of births in the US, with a CB rate of 75% [18]. The vast majority of triplet and higher order gestations are delivered by CB [19, 20]. As most breech and multiple gestation babies are delivered by CB, fewer attendants are skilled in assisting vaginal breech or multiple gestation births, perpetuating a cycle favoring CB. Recently, maternal request has emerged as a CB indication due to tokophobia (fear of birth), previous physically or psychologically traumatic birth, or to avoid perineal injury which may predispose to sexual dysfunction or incontinence [21]. Smaller family size [22] contributes to the concept of ‘precious pregnancies’ for which labor and VB may be considered to pose avoidable risk [23]. Increased safety and prevalence of surgery in general and CB in particular, concern about the uncertainty involved in the onset and course of labor, as well as the perception of some persons that CB is safer than VB for the mother and the baby contribute to the choice of CB [24].

Increased intra- and inter-national mobility alters formerly traditional life ways and sources of physical, economic, and social support. Birth in a hospital attended by intimate strangers poses an evolutionary mismatch compared to the emotionally supportive social setting in which childbirth occurred until quite recently [25, 26]. Participation in the workforce and childcare responsibilities may press pregnant women to seek temporal control of birth; professional staffing schedules of health care workers and facilities benefit from planned deliveries. Although many women indicate a desire for naturalistic birth practices such as non-pharmaceutical pain management, recourse to technologic intervention is common and naturalized, whereby inhaled nitrous oxide, intravenous medication, and epidural anesthetic are viewed as part of the ‘normal’ course of events [27]. Litigation of unfortunate obstetric outcomes, whether due to birth attendant error or to incompletely understood intrauterine processes that produced the perceived anomaly antenatally, or due to a combination of these, generally rewards intervention including CB [28].

Some of the biosocial factors linked to CB include socio-economic status [29], race and ethnicity [30], private versus public payor status [31], care provided by physicians versus midwives [32], time of day [33], day of week [34], and culturally or personally auspicious birth days [35]. Obstetric factors contributing to CB include multiple gestation [36], previous CB [37], and placental abnormalities [38]—the latter two conditions consequent to primary CB. Concomitant demographic trends include advancing age of mothers at first birth [39] which is positively associated with use of assisted reproduction technologies (ART) [40]. ART, in turn, is associated with higher rates of multiple gestation and corollary higher CB incidence [41]. Global health trends of increasing prevalence of maternal obesity, diabetes, hypertensive and cardiac disorders [42] are each positively associated with maternal age and CB incidence [43] as well as with increased risk of complications related to CB itself. Conditions that predispose to CB cluster due to social, environmental, and biological factors.

Familial clustering of CB is a little-studied bio-social phenomenon which may contribute to the normalization of the practice within familial lineages and social networks, increasing the expectation, acceptance, and even preference for CB across generations. Self-reinforcing transgenerational birth practices have profound implications for the future of human reproduction, including differential individual and inclusive reproductive fitness. Adaption to, and reliance upon biomedical technology to effect birth carries susceptibility to increased mortality and morbidity should access to those technologies be disrupted. This study is designed to explore transgenerational trends in birth mode in the United States. A secondary aim is to assess the relationship between birth mode and parity.

## METHODS/METHODOLOGY

This study utilizes mixed methodology of collecting and analyzing narratives of birth mothers. Persons at least 18 years of age who gave birth to at least one live-born child in the United States were recruited to participate in this study which was conducted in compliance with the Institutional Review Board of Wayne State University (Detroit, Michigan). Participants were recruited by snow-ball method, through community notices, and online postings. Trained researchers from diverse social locations accessed a variety of participant populations. Each respondent anonymously provided their own birth history, as well as those of their female maternal relatives: maternal grandmother, mother, sisters with the same mother, and daughters. Opportunity for open-ended responses provided additional qualitative data. Participant interviews were conducted in-person, via telephone and video. An online survey tool was added to facilitate data organization [44].

An earlier qualitative study by the author explored pregnancy and birth experience of 22 first-time mothers, finding that of the 6 women experiencing CB, 4 were themselves born through CB; a fifth was born vaginally, but her mother experienced additional CB’s, leaving only 1 of 6 persons experiencing CB having been born to a mother who was CB-naïve [27]. Power analysis for sample size was informed by this data and indicated that to detect a clinically significant 30% difference in the primary outcome of any CB versus all VB with 0.05 alpha error at 80% power, 76 cases were needed. Twenty-five percent oversampling was planned.

Data including maternal race, education, and family income were provided by participant self-report. African American includes persons who identify as African American or Black. European American includes persons identifying as European American, White, or Caucasian. Hispanic includes persons identifying as Hispanic or Latinx. This study was conducted in a region that is home to many persons originating from the Middle East and North Africa (MENA Region including Lebanon, Egypt, Iran, Iraq, Israel, Morocco, Syria, and Yemen) preferring MENA designation over White as categorized by the US Census Bureau [45]. Infant weights and dates of birth, indications for CB, medical diagnoses, and interventions are per participant recall. Where more than one indication for operative delivery was given, the primary indication (per participant) is reported here. All persons participating in this study identified themselves as women and such terminology is generally used in this work. The questionnaire used for this study is available at www.anthropocenelife.net; original data is available from the author upon request.

Participant data was analyzed by birth year cohort: Baby Boom Generation (BBG) 1944–1964; Generation X (GX) 1965–1980; Millennial Generation (MG) 1981–1996; Generation Z (GZ) 1997–2012. Birth year cohorts were chosen to underscore the contribution of sociality to birth practice; birth year cohorts experienced similar technological changes and cultural zeitgeists. Data were then analyzed by family generation: Grandmother, Mother, Index, Daughter. BBG and GX, having completed their families, were combined to form the Index Generation. MG and GZ were incorporated into the Daughter generation; their mothers joined the Index generation, and their grandmothers joined the Mother generation. Generation time is defined as the average midpoint between maternal age at first birth and at last birth. Fisher Mid-P exact test, Mann–Whitney U-test, and Chi Square test were applied as appropriate, significance threshold *P* value <.05. Relative risk was calculated with a 95% confidence interval, z-score = 1.959.

**Table 1:** Select participant characteristics.

**ALL (n-107)**		**Mean**	**Min.**	**Max.**	**StD**									
**Participant Birth Year**		1971.8	1944	2002	14.2									
**Age at Study**		49.3	21	80	14.2									
**Maternal Age at Birth of First Child (years)**		27.84	16	40	5.04									
US Population*		27.4												
**First Child Birth Year**		2002	1967	2023	14.17									
**First Child Birthweight (g)**		3331	1956	4734	448									
**Education**	**No Answer**		**Grade School**		**High School Grad**		**College Grad or Higher**							
	**n**	**%**	**n**	**%**	**n**	**%**	**n**	**%**						
Study Participants	1	0.9	2	1.9	31	29	73	68.2						
US Population**			9.9%		54.8%		34.3%							
**Household Income**	**No Answer**		**<$50 000**		**$50000-150 000**		**>150 000**							
Study Participants	7	6.54	13	12.15	52	48.6	35	32.71						
US Population**			$74 149											
**Race/Ethnicity/Heritage**	**No Answer**		**African**		**Asian**		**European**		**Hispanic**		**Middle East/North Africa**		**Pacific Islander**	
Study Participants	0	0	11	10.3	4	3.7	75	70.1	6	5.6	10	9.4	1	0.9
US Population***			13.7%		6.4%		75.3%		19.5%		Not Recognized		0.3%	
**Birthplace**	**US Midwest**		**US Southwest**		**US East**		**US West**		**US South**		**US Northwest, Pacific**		**Non-US**	
Study Participants	73	68.2	7	6.5	6	5.6	5	4.7	3	2.8	2	1.9	11	10.3

*Osterman, et al. 2024. ** United States Census Bureau Data, 2018-2022. ***United States Census Bureau

**Table 2:** Parity and Mode of Birth for study participants.

**Parity**		**n**	**%**	**Mean**	**Min**	**Max**	**StD**	**p-value**					
**All Participants**		107	100	2.14	1	5	0.94						
**Birth Mode**	All Vaginal	63	58.89	2.14	1	5	0.93	Ref.					
	All Caesarean	28	26.17	1.79	1	3	0.93	.03*					
	Vaginal and Caesarean	16	15	2.75	2	5	1.13	.01*					
	Any Caesarean	44	41.12	2.14	1	5	0.95	NS					
**Uniparous Participants**		24	22.42	1	1	1							
**Birth Mode**	Vaginal	9	8.41	1	1	1		.04**					
	Caesarean	15	14.02	1	1	1							
**Births By Parity**		**1**		**2**		**3**		**4**		**5**		**All Births**	
		n	%	n	%	n	%	n	%	n	%	n	%
**Total Births**		107	100	83	77.57	27	25.23	8	7.48	4	3.74	229	100
**Vaginal**	Total	72	67.29	57	68.67	21	77.78	6	75	2	50	158	69.00
	Spontaneous	63	58.88	57	68.67	21	77.78	6	75	2	50	149	65.07
	Forceps or Vacuum	3, 6	8.84	0	0	0	0	0	0	0	0	9	3.93
**Caesarean**	Total	35	32.71	26	31.33	6	22.22	2	25	2	2	71	31.00
	Primary	35	32.71	7	8.43	2	7.41	0	0	0	0	44	19.21
	Secondary	0	0	19	22.89	4	14.81	2	25	2	2	27	11.79
**Caesarean Indication**		n	%	n	%	n	%	n	%	n	%	n	%
Failure to progress		16	45.71	2	7.69							18	25.35
Fetal distress		5	14.29	1	3.85							6	8.45
Failed induction		4	11.43									4	5.63
‘Baby too big’		3	8.57	1	3.85							4	5.63
Breech presentation		3	8.57	2	7.69	1	16.67					6	8.45
Elective/by choice		2	5.71									2	2.82
Placenta previa/low		1	2.86									1	1.41
Multiple gestation		1	2.86	1	3.85							2	2.81
Fetal condition						1	16.67					1	1.41
Failed labor trial, previous caesarean				3	11.54	1	16.67	1	50	1	50	6	8.45
Elective repeat caesarean				16	44.67	3	50	1	50	1	50	21	29.58

* Difference of means. ** Difference of proportion

### Study population characteristics

A total of 118 responses were collected between June, 2023 through January, 2024; 11 were excluded due to incomplete data, leaving 107 cases for analysis. Select participant characteristics are described in Table 1. US reference data is provided for context [46]. Participants ranged widely in age (21–80 years, mean 49.3, std 14.2); the mean birth year of participants was 1973.8 (1944–2022, std 13.9). First birth occurred at a mean maternal age of 27.8 years (range 16–40, std 5.15), comparable to the national average of 27.4 years [2]. The majority of participants were US-born (*n* = 96, 89.7%), approximate to US Census data (86.3%) [46]. Eleven participants (10.3%) were foreign-born: seven in MENA countries, others in East Asia, India, East Africa, Eastern Europe, and Mexico. Although most subjects gave birth in the Midwest, all US regions are represented. The sample was comprised of persons identifying as African American or Black: *n* = 11 (10.3%); Asian American: *n* = 4 (3.7%); European American or White: *n* = 75 (70.1%); Hispanic American or Latinx: *n* = 6 (5.6%); Middle Eastern/North African (MENA): *n* = 10 (9.4%); and Pacific Islander: *n* = 1 (0.9%). Study participants were more highly educated than reported US averages: 68.2% of study participants achieved a bachelor’s degree or higher compared to the US average of 34.3%. The mode of annual household income in this study was $50–150 000, comparable to the national average of $75 149.

### Study participant birth outcomes

Mean singleton birthweight was 3331 g (1956-4734 g, std 448 g); singleton birthweight did not differ significantly between babies born vaginally and via CB (3309 g, std 406 versus 3413 g, std 502; *P* = 0.25). Multiple gestation occurred in 2 of 107 first pregnancies: 1 set of twins and 1 set of triplets (triplets achieved through ART). Birth mode and parity data for study participants are presented in Table 2. Of first births, 35 (32.7%) were CB and 72 (67.3%) were VB. Three VB were assisted with forceps and 6 with vacuum; no participant reported forceps or vacuum use other than for the first birth. The most common indication for CB was failure to progress in labor (*n* = 16, 45.71%), followed by fetal distress or ‘concern for baby’s heart rate’ (*n* = 5, 14.29%), failed induction of labor (*n* = 4, 11.43%), ‘baby was too big’ (*n* = 3, 8.57%), breech presentation (*n* = 3, 8.57%), elective (*n* = 2, 5.71%), abnormal placental location (*n* = 1, 2.86%), and multiple gestation (*n* = 1, 2.86%). The ‘any CB per birthing person’ metric reveals that the experience of any CB is more common (44/107, 41.12%) than the total per-birth CB rate (71/229, 31.00%; *P* = .03) or the primary CB rate (44/229 total births, 19.21%; *P* = .00001) indicate, and trends higher than the first-birth CB rate (35/107, 32.71%; *P* = .10). Although the highest rate of primary CB occurred with the first birth, primary CB also occurred for second (7/83, 8.43%) and third (2/27, 7.41%) births.

The average parity of study participants was 2.14 (range 1–5). No one whose first birth was CB delivered more than four children; no individual in this study experienced more than three CB. Although women who experienced all VB had higher parity than those experiencing all CB (2.14, range 1–5 versus 1.79, range 1–3, *P* = .03), those with any CB had highest parity (2.75, range 2–5, *P* = .01). Taken together, there was no difference in parity between persons experiencing all VB compared to those having experienced any CB, as this latter group includes both mothers with highest parity and those experiencing a single CB. Of persons who gave birth to a single child, a greater proportion experienced CB than VB (15/24 versus 9/24, *P* = .04). Participants reported major morbidity after both VB (seven recto-vaginal tears, one with bleeding requiring transfusion) and CB (one bleeding requiring transfusion with delivery of triplets). No participant related having experienced hysterectomy or neonatal death.

**Table 3:** Birth year cohort and Birth Mode.

**Total Participants n = 107**	**Cohort Birth Years**	**n (%)**	**Birth of Participant (n, %)**		**Participant’s First Birth**				**All Births to Participant**		**US Reference**	
			**Via Caesarean**	**Participant Mother, any caesarean**	**Mean Year (range)**	**Age (years)**	**Birth Weight (g)**	**Caesarean n (%)**	**Mean Parity**	**Any Caesarean n (%)**	**Year**	**Caesarean Rate (%)**
**Baby Boom Generation**	1944–1964	31 (29.0)	1 (3.23)	2 (6.45)	1985 (1967–1996)	28.03	3337	7 (22.58)	2.32	11 (35.48)	1985 ^27^	22.7
**Generation X**	1965–1980	41 (38.3)	5 (12.20)	11 (26.83)^	2002 (1989–2019)	29.02	3382	15 (36.59)	2.27	18 (43.90)	2002 ^28^	26.1
**Millennial Generation**	1981–1996	30 (28.0)	3 (10.00)	9 (30.00)	2016 (2003–2022)	27.2	3266	11 (36.67)	1.93*	13 (43.33)*	2016 ^29^	31.9
**Generation Z**	1997–2021	5 (4.7)	1 (20.00)	2 (40.00)	2020 (2018–2023)	20.8	3549	1 (20)	1.20*	2 (40.00)*	2020 ^30^	31.8

*Millennial Generation and Generation Z may not have completed childbearing

^Difference of Proportion p = 0.03.

### Analysis by participant birth year cohort

Participant birth year cohort and mode of birth data are presented in Table 3: BBG; GX; MG; and GZ. GX participants comprised the largest cohort (*n* = 41, 38.32%) followed by BBG (n = 31, 28.97%), MG (n = 30, 28.04%), and GZ (n = 5, 4.67%). Mean year of first birth was as follows: BBG 1985, GX 2002, MG 2016, GZ 2020. No difference was found in the CB rate between birth year cohorts. Accordingly, no difference in the proportion of individual women who experienced any CB birth was identified between birth year cohorts for: BBG (11/31, 35.48%), GX (18/41, 43.90%), MG (13/30, 43.33%), GZ (2/5, 40%) (p = 0.68). US population reference data is provided for context [47–50]. Note that births to most study participants occurred after CB rates had already increased dramatically in the US [47]. No birth cohort difference was identified for persons who were themselves born by CB (*P* = .51). The proportion of persons whose mothers experienced any CB compared to all VB approaches statistical significance (*P* = .08): BBG (2/31, 6.45%), GX (11/41, 26.83%), MG (9/30, 30.00%), GZ (2/5, 40%). No difference was found between first-born birthweights for any birth cohort (BBG 3336 g, GX 3382 g, MG 3266 g, GZ 3549 g; *P* = .31). No difference was found in parity between BBG (2.32) and GX (2.27) (p = 0.40). Maternal generation time for BBG and GX study participants was calculated as 30.5 years. MG and GZ participant data were not included in parity and generation time calculations as these women may not have completed childbearing.

### Analysis by generations of family lineage

Individual family generations were then compared: Grandmother, Mother, Index, and Daughter. BBG and GX form the Index generation as they have completed childbearing. Data from 35 MG and GZ participants, all of whom were born to BBG or GX mothers, contributed to the Daughter generation; their BBG and GX mothers were incorporated into the Index generation; MG and GZ grandmothers were incorporated into the Mother generation. In five cases, an MG or GZ participant was born outside of the US and their case was removed from analysis as this study is limited to the narratives of participants who themselves experienced at least one birth in the US, leaving 102 lineages for analysis. An intergenerational comparison of parity and mode of birth is presented in Table 4. Note that generational cohorts vary in size due to limited knowledge or recall of Grandmother experience, inclusion of MG, GZ participants in Daughter generation, and the limited number of parous Daughters of Index.

**Table 4:** Intergenerational comparison of Parity and Birth Mode by maternal lineage.

**GENERATION**		**Grandmother of Index**		**Mother of Index**		**Index**		**Daughters of Index**		**Individuals of all Generations**	
**n**		69		102		102		49		322	
**Birth Year (Range)**		1912 (1880–1936)		1939 (1909–1964)		1965 (1944–1980)		1989 (1983–2002)			
**PARITY mean (Range)**		4.25	(1–12)	3.54^	(1–7+)	2.43^^	(1–5)	Families Incomplete			
	**Any Parous Daughter**	69		102		49		0			
	**Parity Difference from Preceding Generation**			^p = 0.01		^^p = <.00001					
**BIRTH MODE**		**n**	**%**	**n**	**%**	**n**	**%**	**n**	**%**	**n**	**%**
**All Vaginal Birth**		**68**	**98.55**	**81**	**79.41**	**65**	**63.73**	**27**	**55.1**	**241**	**74.84**
	**Parous Daughters**									182	
	**All Vaginal Births**	56	82.35	49	60.49	20	60.61	0		125	68.68
	**Any Cesarean**	12	17.65	32	39.51	13	39.39	0		57	65.79
**Any Cesarean Birth**		**1**	**1.45**	**21**	**20.59**	**37**	**36.63**	**22**	**44.9**	**81**	**25.16**
	**Parous Daughters**									38	
	**All Vaginal Births**	0	0	6	28.57	7	43.75	0		13	34.21
	**Any Caesarean**	1	100	15	71.43	9	56.25	0		25	65.79
**Parous Daughters Birth Mode Difference**				p = 0.01		p = 0.36		No Parous Offspring		p = 0.0002	
**Relative Risk of Caesarean, Mother Any Caesarean**				1.81 (1.23–2.65)		1.43 (0.78–2.60)				2.1 (1.53–2.88)	

The incidence of any CB to individual birthing persons increased across generations: Grandmother (1/69, 1.45%), Mother (21/102, 20.59%), Index (37/102, 36.63%), Daughter (22/49, 44.9%) (*P* ≤ .00001). The incidence of CB to a daughter of a person who themself experienced CB is increased compared to the incidence of CB in daughters of persons who experienced all VB (*P* = .0002). The overall relative risk for a person who has experienced any CB to have offspring who also have CB is 2.1 (1.53–2.88).

## DISCUSSION

This report offers three contributions to the discussion of changing patterns of human birth. Firstly, CB is more common within family lineages: CB is more likely among daughters born to mothers who themselves experienced CB. CB clustering by family lineage indicates that that surgical birth, or the biosocial factors that result in surgical birth, predispose future generations to the same. This study includes CB for any indication, as per maternal report: it is not limited to those related to FPD or to the more nebulous categories of failure to progress in labor, non-progressive labor, failed labor induction, or simply ‘baby was too big’. Even so, the results of approximately two-fold increased risk ratio are in keeping with estimations based on modeling [7]. A Scandinavian population-based study found increased risk of CB for FPD between mothers and eldest daughters (Odds ratio 1.7, CI 1.2–1.4) [51], and another identified increased risk of first-birth CB for any reason to a mother who was herself born by CB (RR 1.5, CI 1.48–1.62) and for younger sisters of older sisters who had CB (RR 1.45, CI 1.40–1.51) [52]. A recent population-based Canadian study found an increased risk of CB in first births to women who were themselves delivered by CB (RR 1.36, CI 1.30–1.43) [53]. The current study differs in that it is an interview-based analysis of histories reporting births to four maternal generations (Grandmother, Mother, Index, Daughter generations). Rather than estimating risk of CB for the first birth to a person born by CB, it includes risk for any CB to individual women through their lives.

Secondarily, this study reports individual lifetime CB incidence in addition to the commonly reported CB rate for first birth, CB rate for individual births, and the primary CB rate. The ‘any CB per birthing woman’ metric reveals that CB experience is more prevalent within the population than other common measurements indicate. The CB rate is highest for first births in this analysis, as has extensively been reported elsewhere [54]. Although electively scheduled repeat CB is the most common indication for CB of subsequent children, primary CB continues to occur at greater parity for indications such as failure to progress in labor, multiple gestation, fetal distress, breech presentation, and by maternal choice. As the prevalence of CB increases during individual lives and over generations, the pool of persons born to mothers who have experienced CB also increases. Increasing familiarity with CB within families, social spheres, and across greater society normalizes CB as a birth option or as a possible expectation. ‘Everyone in my family has c-sections’ is a quote from a study participant who came to this understanding within just 3 generations of caesarean being a largely perimortem procedure. The ‘any cesarean’ per birthing woman metric is valuable in assessing the impact of birth mode on individuals, families, and populations. Larger cohorts including birth outcomes for entire maternal reproductive careers, including CB’s and indications, would assist in understanding the role of family experience in birth mode expectation and outcomes.

Thirdly, the relationship between CB and parity is complex. Earlier work identifying an association between CB and smaller family size implicates both physiological factors and intentional pregnancy avoidance [55]. In this study, we hypothesized that parity would be lower for persons who experienced CB, possibly due to concern for known complications such as risk of uterine scar disruption, placental implantation abnormalities, maternal conditions associated with the first CB, previous adverse birth experience, and/or ease of permanent sterilization at the time of surgery. Persons who experienced all CB did have lower parity than those who experienced all VB. However, persons who had any CB (both VB and CB) had the highest parity of the three groups, possibly due to desire for a larger family, greater willingness to attempt a trial of labor after previous CB, and greater success in doing so. Hence, we found no overall parity difference between persons experiencing all VB compared to any CB. Of the four persons in this study of highest parity (parity = 5), two had all VB; the remaining two had initial VB’s and final CBs with one or two intervening CB’s. Persons with 1 child were more likely to have experienced CB than VB; none of these persons indicated that the CB was elective.

Declining birthrates in the US and worldwide have recently come under scrutiny [56, 57]. Primary CB carries risks for surgical complications in later pregnancies that increase in proportion to the number of CB an individual experiences [58]. Permanent sterilization by tubal ligation is easily accomplished after CB of the baby and may factor into individual decisions for CB [59]. In a reported survey representing 20% of US hospital births between 2016 and 2018, Fan and Westerhoff found that women undergoing CB had higher odds of having a tubal ligation than women undergoing VB (8.83; CI 8.73–8.97) [60]. Thus, CB may contribute to smaller family size, limiting reproductive fitness. This study, however, found highest parity for women experiencing both CB and VB. Longitudinal studies of larger sample size of persons from diverse backgrounds including communities traditionally valuing high parity in a study specifically designed to explore the relationship between CB and parity would inform understanding of the role of CB in family formation.

### Study limitations

This study has several limitations, one being its relatively small sample size, another being the long human generation time (30.5 years in this study). Persons in younger birth year cohorts (MG, GZ) or in latter generations (Daughter generation) may not have completed or even started their families. Despite the rapidity with which CB has emerged as a common procedure, more time is needed to observe its impact. However, the primary aim of this study, analysis by family generation linking individuals to their direct maternal relatives, is informative and supports the notion that CB is more common in lineages where it has already occurred. Although efforts were made to include participants from a breath of backgrounds, our population characteristics differ from other US and world populations limiting generalizability. Larger numbers of family lineages tracing birth mode and parity in additional settings are needed to address the robustness of these findings.

This study relies on maternal knowledge, recall and willingness to anonymously share the life events of self and family members. Narratives are related in lay terms from the perspective of individual mothers and reflect their understanding of their own and others’ histories. Many study participants had limited knowledge of their mothers’ birth experiences and often knew very little about their grandmothers. Although researchers were trained in nondirective, non-judgemental interview techniques, persons may be reluctant to share information that they do not wish to recall or narratives that they feel may be received unfavorably such as children who died or were given for adoption, complications, or reasons for CB (e.g. maternal choice, active herpes infection). Knowledge of forceps or vacuum deployment, especially for persons other than self was particularly problematic as heavy sedation compromising recall including ‘twilight sleep’ was formerly common [61]: ‘the baby was handed to me later, wrapped in a blanket’ (quote from study participant). Thus, forceps and vacuum use were not accurately determined. Such interventive birth practices, potentially naturalized by family experience or possibly by other biosocial factors may connote predisposition to require, request, or accept technologic solutions including CB, even as forceps and vacuum use declined. Review of medical records triangulating technology use, delivery mode, and birth outcomes with provider and birthing women’s perspectives would provide additional insight and data robustness.

### Birth as a biosocial process

Birth is a biosocial process. In humans, successful reproduction has been shaped by functional and physical advantages and constraints including those associated with bipedality and encephalization, and by our sociality [ 62]. Humans have been described as cooperative breeders [63] and as practicing ‘biocultural reproduction’ [64]. The tragedy of maternal and perinatal mortality and morbidity are ongoing aspects of human history mitigated by social technologies of birth assistance, which may predate our species [65]. Biomedical technological advances such as antibiotic therapy, blood banking, anesthesia, and surgical techniques including CB further improve both mother and child outcomes. The fetus became an entity of consideration separate from the mother in the late 1900’s with the development of obstetric ultrasound, fetal heart rate monitoring, and other testing modalities sometimes prompting intervention by CB, variably contributing to improvements in neonatal mortality and morbidity. Production of a human child is thus a biosocial endeavor.

The optimal CB rate is that at which complications are outweighed by benefits as compared to VB for mother and child. Adverse events may arise from intervention occurring both ‘too little, too late’ and ‘too much, too soon’ [ 66]. Evidence-based obstetric practice continues to incorporate objective parameters by which intervention is deemed warranted [67]. For example, the Robson criteria for classifying CB indications [68] has been implemented around the world in an effort to standardize reporting and to facilitate outcome comparison, with varying results [69]. However, the decision for CB is distributed between the birthing woman, their care provider(s), and other agents. Online sources including social media have emerged as important fonts of information and support accessed by pregnant women [27]. Rapid changes in technical, biological and social factors over recent generations are reflected in altered childbirth practice. The increased incidence of any CB to individual women for a composite of indications within maternal lineages identified by this study suggests that the ideal CB rate is an upwardly moving target.

## CONCLUSION

Within two to three generations, humans have embraced surgical birth such that CB is now the dominant mode of delivery in some countries [70]. Potentially heritable anatomic changes due to liberal CB use include lack of selection for maternal pelvic or neonatal head size and physiologic change due to avoidance of the cascade of events that characterize unmedicated VB. Evolutionary mismatches between ancestral and modern diet and lifestyle contribute to indications for CB and consequently to its incidence. The ‘cliff-edge’ may be removed by CB [7], but simultaneously, a multitude of other ‘injurious variations <are no longer> rigidly destroyed’ [6]. Biosocial technologies also pose mismatches contributing to CB: medical care allows people with serious conditions to successfully reproduce, infertility may be overcome with ART, effective contraception facilitates older mothers at first birth and lower parity, vaccination and improved nutrition increase infant and child survival, ultrasound and other in-utero assessments promote the treatment of fetuses as family members, human lives are constructed as separate from the broader natural world. Each condition was absent in our ancestral environment. We are super social mammals; widespread social networks and media promulgate ideas of modernity, beauty, health, and relationships. Technologies of monitoring self and others, including menstrual cycles, pregnancy, birth and child-rearing, construct a panopticon of our collective creation [71] which informs our technology use and naturalizes its incorporation in how we view and enact our biosocial selves.

A comprehensive comparison of the long- and short-term risks and benefits of CB versus VB for mother, children, and kin as well as potential proximate and ultimate evolutionary sequelae are beyond the scope of this manuscript. CB saves the lives of some mothers and babies. Devastating consequences of obstructed labor, including maternal and perinatal death, obstetric fistula and disability continue to occur worldwide, especially where access to skilled care is inaccessible [72]. However, quantifying the role of CB is difficult as direct comparisons of different human populations and across time are obfuscated by numerous variables. In settings with access to quality obstetric care, planned, pre-labor CB in healthy women carries similar risk of death and major morbidity as planned VB, planned VB ending in urgent or emergent CB during labor carries greatest risk, while planned VB resulting in successful VB presents least risk to mother and child [73]. Overall, successful VB carries less risk of serious complications than CB. However, CB is more common when medical or obstetric complications pre-exist or arise during pregnancy and labor. The myriad of associated biosocial factors previously described attest to the difficulties in determining when CB is objectively indicated, requiring valuation of others’ risk/benefit determinations and motivations.

Evolutionary trade-offs for progeny due to CB are also difficult to tease out. For example, CB is reported to be associated with an increased risk of food allergies [74]. However, CB is also associated with a suite of biological and social conditions including an altered neonatal microbiome [75], lower rates and duration of breast feeding [76], which is associated with early introduction of solid foods [77], which in turn may predispose the child to obesity, diabetes, and metabolic syndrome later in life [78]. Each of the latter conditions are associated with increased risk of infertility in adulthood, which may be addressed with ART and accompanied by more CB. Obesity and diabetes are known to cluster within families due to learned dietary patterns [79], also predisposing progeny to require CB.

Implications of abdominal birth or avoidance of gestation in maternal bodies altogether have been explored through film and literature, often focusing on selection for large heads, of bio-socially desired characteristics, and of dualistic access to medical care [80, 81]. Recent speculation that vaginally-born babies’ brains are restricted in size is seriously misplaced [82]. There is no evidence that VB stunts or CB promotes brain development. Increasingly complex tool use, language, abstract thinking, and the associated increased encephalization which have characterized our genus may continue; this trend may be facilitated by CB. However, evolutionary pressures must be exerted for generations before differences emerge, influenced by strength of positive and negative selective pressures. An immediate concern for increasing recourse to and dependence on surgical birth is that access to quality care may be limited due to disparities between or within populations or may occur when access to care is broadly disrupted due to natural or human-precipitated disaster. Interrupted access to surgical birth after a period of adaptation to and reliance upon biomedical technologies may be associated with increased maternal and infant morbidity and mortality as compared to populations reliant on VB.

The most important evolutionary mismatch related to CB may be that a technology developed to improve maternal and child outcomes may predispose lineages that come to rely on operative birth to leave fewer progeny. Over generations, such lineages diminish in the population. Although it is intuitive that some societies value large families more than others, rapidly changing global fertility patterns attest to widespread, profound changes in family structure. The relationship between parity and CB within lineages was not clear from this work; larger studies of diverse populations over coming generations may shed light on this important issue.

Reliable, equitable access to skilled, compassionate maternity care and to judicious use of technologies including caesarean are critical to ensuring that all persons benefit from their appropriate application. The impact of caesarean birth on the trajectory of *Homo sapiens* will be known over coming generations—too long for one observer stationary in the Anthropocene, but just a moment of evolutionary time.

## Suggested Referees

1. Wenda Trevathan PhD

New Mexico State University (Emeritus).

wtrevath@nmsu.edu

1. Karen Rosenberg PhD

University of Delaware.

krr@udel.edu

## Acknowegements

This work was supported in part by a grant from Wayne State University.
